# Identification and correction of previously unreported spatial phenomena using raw Illumina BeadArray data

**DOI:** 10.1186/1471-2105-11-208

**Published:** 2010-04-27

**Authors:** Mike L Smith, Mark J Dunning, Simon Tavaré, Andy G Lynch

**Affiliations:** 1 Cancer Research UK, Cambridge Research Institute, Li Ka Shing Centre, Robinson Way, Cambridge, CB2 0RE, UK

## Abstract

**Background:**

A key stage for all microarray analyses is the extraction of feature-intensities from an image. If this step goes wrong, then subsequent preprocessing and processing stages will stand little chance of rectifying the matter. Illumina employ random construction of their BeadArrays, making feature-intensity extraction even more important for the Illumina platform than for other technologies. In this paper we show that using raw Illumina data it is possible to identify, control, and perhaps correct for a range of spatial-related phenomena that affect feature-intensity extraction.

**Results:**

We note that feature intensities can be unnaturally high when in the proximity of a number of phenomena relating either to the images themselves or to the layout of the beads on an array. Additionally we note that beads neighbour beads of the same type more often than one might expect, which may cause concern in some models of hybridization. We highlight issues in the identification of a bead's location, and in particular how this both affects and is affected by its intensity. Finally we show that beads can be wrongly identified in the image on either a local or array-wide scale, with obvious implications for data quality.

**Conclusions:**

The image processing issues identified will often pass unnoticed by an analysis of the standard data returned from an experiment. We detail some simple diagnostics that can be implemented to identify problems of this nature, and outline approaches to correcting for such problems. These approaches require access to the raw data from the arrays, not just the summarized data usually returned, making the acquisition of such raw data highly desirable.

## Background

A key stage for all microarray analyses is the extraction of feature-intensities from an image. If this step goes wrong, then the experiment will certainly be compromised. Thus, much research has gone into the tasks of automatic identification of both the features on arrays and the grids on which they lie [1-4]. Illumina BeadArrays differ from other types of microarray in their construction, and have had special attention paid to them as a consequence [5,6].

Illumina employ random construction of their arrays on a hexagonal grid [7]. As a consequence, each probe will occur a random number of times on the array and in random locations. This means that the beads have to be found twice with Illumina BeadArrays (thrice with two-colour versions): once by Illumina to identify the type of bead present at a location, and once (or twice) by the user to quantify intensities after hybridization. These steps are then even more important for the Illumina platform than they are for other technologies.

While Illumina's software can report the raw bead-level data from an array (i.e. the location and intensity for every bead), more typically only summarized data are produced. We have previously shown that there are advantages to working with the bead-level data [8], not least the abilities to calculate covariances in two colour platforms [9] and to identify and correct spatial artefacts [10]. In this paper we show that using the bead-level data it is possible to identify, control, and perhaps correct for a range of other phenomena related to the locations of the beads. In particular, we will consider a number of potential effects that are suggested by previous studies.

For example, it has been shown [11] for microarrays that the depletion of target molecules, due to those molecules hybridizing to probes, can affect the physical chemistry to an extent that the registered intensity will also be affected. Such effects can occur locally, and the nature of the Illumina array means beads hybridizing to the same target may be proximal, in which case concerns about local depletion may be raised. A previous study [10] showed anecdotal evidence that clusters of beads that failed to be decoded were spatially associated with regions of outliers, suggesting that such regions might be beneficially excluded.

Other theoretical aspects of the technology require investigation. The background intensity for arrays is calculated in a non-robust manner that might lead to extreme and biased values. Additionally, the high-density structure of the array may lead to situations where the intensity of a bead may influence that of its neighbours.

There are three key steps in moving from an image of a BeadArray to a set of intensities. First the bead locations must be identified, second the beads must be mapped to a bead-type, and third, an intensity value extracted. Here we investigate each of these steps, showing how each can bias the final intensity, but showing also how such errors can be identified and corrected when working with bead-level data. We illustrate this using two bead-level data sets (one single-colour expression, one two-colour genotyping/DNA copy number).

## Methods

### The physical arrays

**Figure 1:**
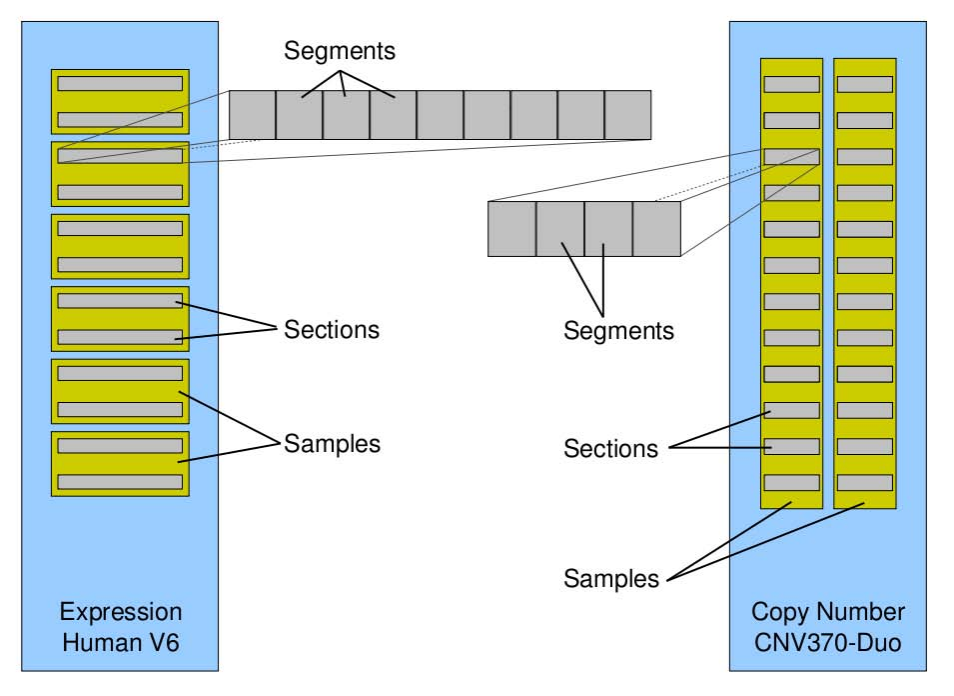
**Illumina BeadArray structure**. Depicting, approximately to scale, the structures of the two types of BeadArray used in this manuscript, and illustrating the terminology we employ for describing BeadArrays. Indicated, for the Human-WG6 V2 expression array and the CNV370-duo DNA copy number array, are the layouts of samples and sections. Additionally, for each technology, one section is expanded to illustrate the segments that comprise it. The images returned as part of the raw Illumina data are of sections, while registration takes place by the segment.

Here we use two Illumina BeadArray platforms: The Human-WG6 V2 single-channel expression array, and the CNV370-duo two-channel genotyping and DNA copy number platform. Their differing structures are depicted in Figure 1, to allow for comparison and to clarify terminology. The expression array accommodates 6 samples, with two array sections devoted to each sample and each section divided into 9 segments. By contrast, the CNV370-duo array holds only 2 samples but has 12 sections per sample, with each section divided into 4 segments. These fundamental segments also differ between the two technologies, with 129, 422 beads (326 × 397) on the expression array, and 147, 519 beads (333 × 443) on the copy-number array.

### Data sets

The copy number data we use consist of two arrays from a previously published data set, full details of which are provided by Curtis *et al *[12]. The arrays we consider have IDs 4127130020 and 4127130188, to which were hybridized a tumour sample and HapMap CEPH NA10851 cell line respectively. The expression data set consists of two chips (IDs 4343238066 and 4343238080) that have been hybridized with two common commercial reference RNA sources (Stratagene Universal Human Reference RNA, and the Ambion Human Brain Reference Total RNA). Since public repositories are not designed to deal with the raw Illumina data that we use here, these data are available to download from our website [13].

The raw data we consider to be the .*tif *image file arising from the scanned array, the .*txt *file containing the locations of all decoded beads (decoding is discussed in the next section) as well as the associated (background-corrected) intensities, the .*locs *file that additionally contains the locations of the beads that were not decoded, and the .*sdf *file that contains details of the chip structure. To reduce download sizes the .*txt *and .*locs *file have been compressed using the package *BeadDataPackR *[14]. Instructions for obtaining bead-level data from the scanner can also be found on our website.

### Availability of methods

All analyses were conducted in R, primarily through the *beadarray *package [15]. Where we introduce functionality that is not part of *beadarray *or another BioConductor package, we make this available through an R script containing the additional functions (Additional File 1), and a vignette that will reproduce the figures and tables in this manuscript (Additional File 2).

### Preprocessing

The array having been scanned, we can consider Illumina's preprocessing as consisting of three distinct steps: registration of beads, mapping of bead IDs, and feature-intensity extraction.

The registration process is described in full by Galinsky [5], but can be summarized thus: First, a bead detection algorithm is used to locate bright beads, then based on these positions, the locations of the remaining beads are interpolated. Finally the grid is shifted to ensure it is centred over the array. Due to the random construction, in addition to finding the beads it is necessary to match the beads to their bead-types. Illumina do this, using a hybridization-based procedure (termed decoding) to identify the probe type attached to each bead [16].

The feature-intensity extraction consists of the calculation of background values, the 'sharpening' of the image via a simple filter that compresses observed fluorescence into tighter regions around the features [17] and then the calculation of foreground values. Background values are simply subtracted from foreground values to return the final intensity.

### Bead registration and mapping of bead IDs

Illumina return a list of bead locations that have been mapped to their pre-determined grid of bead identities. Ideally, the bead-locations will form a regular hexagonal grid, and we can identify beads that are distant from their grid position which may indicate registration issues. This we do separately for each segment, using a simple linear model:(1)

where *P*_*x *_and *P*_*y *_are the bead-centre coordinates (in pixels), and *G*_*x *_and *G*_*y *_are the grid locations. The statistic  gives a measure of departure from the grid. Note that for two-colour platforms, the difference between red and green locations will also be an indicator.

Further, we consider the possibility that the mapping of bead identities to the beads can go wrong. This will either occur for a subset of beads, in which case we may detect it using our measure of departure from the grid, or it will occur for all beads, in which case we must remap the grid positions for each bead ( = *G*_*x *_+ *Δ*_*x *_etc.) and use the bead identities associated with these new grid positions. The shift *Δx *can be determined manually, or automatically by finding the shift that minimizes the mean within bead-type variance for the affected segment and maximizes correlation between replicate segments.

### Foreground calculation

A large variety of algorithms have been employed to extract feature-intensity values from scanned microarray images, and their relative merits have been extensively reviewed [18]. Here we consider only Illumina's foreground algorithm [17] which begins with the bead-centre positions that were identified during the registration step. The foreground value is calculated as a weighted average of sharpened intensities from a 4 × 4 pixel square located about the bead-centre. The centre four pixels of this square always take a maximum weighting, but the weights of the remaining pixels are determined by the fractional part of the bead-centre coordinates as illustrated in Additional File 3.

This process relies on being able to identify the centre of a bead to a resolution of a fraction of a pixel. Illumina report the bead locations to between 2 and 4 decimal places of a pixel in the .*txt *file, whilst they are stored as single precision floating point values (7-8 decimal places) within the .*locs *file.

### Background calculation

Background values are calculated from a 17 × 17 square of pixels located about the bead-centre in the non-sharpened image [17]. Thus the background being calculated is local rather than global, but not bead-specific. Within this square, the mean of the five lowest intensities is taken to be the background value for that bead. The mean is not a robust summary of such extreme values, and we consider alternatives through new functionality in the *beadarray *package.

Note that a 17 × 17 square could contain 12 bead-centres (see Additional File 4) and so it is inevitable that beads will share some or all of their five lowest background pixels (or conversely a pixel may contribute to the background of up to 12 beads). It is also clear that many of the pixels in that 17 × 17 square will be contributing to foreground calculations. We mask the 4 × 4 pixel square around each bead-centre that contributes positively to the foreground calculation in order to calculate the number of "true background" pixels a bead actually has out of the 289 considered.

The separate foreground and background values are not returned by Illumina's software, so we have used the *beadarray *package to obtain these values.

### The bead-location/bead-intensity circle

From the details of the foreground calculation, it is clear that the reported bead-intensities are influenced by the precise bead-location. However the registration of bead locations is partially dependent on bead intensities and will influence the precise location of the bead. While in isolation, each of these steps appears sound, the potential for some 'feedback' in this loop (intensity affects location affects intensity) is of concern.

#### Simulation of beads

To investigate the magnitude of the bead location effect, we perform simulations of idealized beads. Each bead on the array we consider to be a sphere evenly covered in probes. This we assume results in a smoothly fluorescing feature when the bead is scanned. Then, we simulate the digitization of the image into discrete pixels by creating a pixel grid, integrating the fluorescence within each square of the grid, and rounding to an integer value. We then systematically move the grid, a fraction of a pixel at a time, and calculate the foreground intensity using the known bead-centre location.

#### Investigation of association in data

We use the fractional part of the bead-centre locations and the intensities obtained from Illumina's .*txt *file to investigate the patterns of association in real data. We break a theoretical pixel into 100 × 100 bins and, for each bin, plot the mean of all log-intensities for beads whose fractional bead-centre locations coincide with the bin.

Where we identify an association, we investigate the nature of any causality by a) recalculating intensities with location-independent weights, and b) by averaging not the log-intensities but the residual log-intensities once the average for that bead-type has been removed. Since beads of the same type should be showing similar intensities, they should be similarly affected by the bead registration algorithm, and so this second step tests the possibility of log-intensity affecting location.

### Identifying beads near phenomena of concern

Clusters of non-decoded beads are identified by finding the locations of all non-decoded beads from the .*locs *files and mapping the neighbours of all beads. We assume that any such cluster of beads will contain a non-decoded bead with six non-decoded neighbours, and each such bead is used as a seed to identify maximal networks of neighbouring non-decoded beads (by use of an invasion process). Clusters we define as being any such network of 50 or more beads, and these are then expanded further to identify successfully decoded beads in the vicinity of the cluster.

To identify bright beads that might interfere with their neighbours, we use the *EBImage *package (version 3.3.1) [19] to segment the image and then select bright features with a mass of at least 40 pixels and a high degree of circularity. Beads with aberrant background calculations are identified using the *readTIFF *function in *beadarray *which allows us to interrogate the pixels surrounding a bead.

Identifying beads that neighbour beads of the same type is trivial using the neighbours matrix. In an idealized array containing 1, 000, 000 beads from 50, 000 bead-types where each bead has six neighbours we would expect to have 60 such pairs. This calculation ignores some of the structure of the array (most obviously that not all beads have six neighbours), and we investigate the numbers that we would expect for our arrays under a truly random construction as follows: We maintain the neighbours matrix for an array, but permute all bead identities of beads that were successfully decoded, tally the number of neighbouring pairs of the same type, and repeat.

### Assessing impact on biological findings

To assess the impact of the identified phenomena we use the *beadarray *package along with the scripts we provide here to identify affected beads and remove them prior to summarization. Analyses follow the scheme illustrated in Additional File 5, and are repeated without adjustment for these phenomena to allow for assessment of their impact.

## Results and Discussion

### Bead-centre location

**Figure 2:**
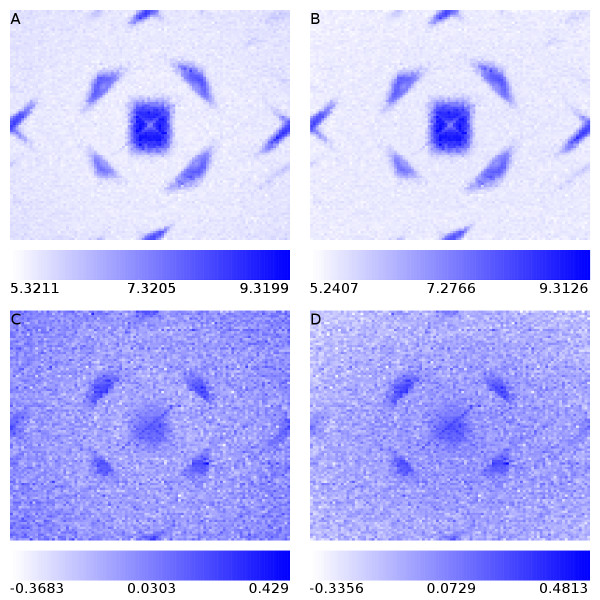
**The relationship between the fractional part of bead position and observed intensity**. (A) To investigate the association between bead-intensity and the fractional part of a bead's location we use the bead coordinates and intensities for array section 4343238066_A_1. A pixel is divided into 100 × 100 bins and the average log-intensity of beads with fractional coordinates falling into those bins has been plotted. (B) As for part A of the figure, but the fractional part of the coordinate was not used in the intensity calculation, with the four pixels instead being given equal weight. (C) As for part A of the figure, but within bead-type residual log-intensities are plotted. (D) As for part B of the figure, but within bead-type residual log-intensities are plotted.

On investigation of the possible dependence between the fractional part of the coordinate, and the derived log-intensity, a striking association is seen (Figure 2). That this largely remains when the fractional coordinates are not used to weight the foreground calculation suggests that the majority of the association is due to intensity driving location rather than the other way about. However, the fact that the association is still strong when plotting within-bead-type residual log-intensities suggests that there may still be an element of location determining intensity.

We conducted simulations to establish whether such a large effect of fractional location on intensity was plausible. Under our simulations, it is clear that the relative position of the pixel grid over the bead can influence the measured intensity, with the value being greatest when the fractional part of the bead-centre coordinates is close to zero. The range of values that can be achieved is small however, being of the order of 0.2 on the log intensity scale for large bright beads. This value increases as the bead image becomes smaller relative to the pixels (Additional File 6), which would correspond to lower intensity beads, for which such a change might be more important. However this effect is the opposite of the association seen in the real data, suggesting once more that intensity determines location more than location determines intensity.

Ostensibly, this broad result is reassuring. If location were determining intensity to a much larger degree than seen here, then this would be a fundamental problem. Yet even if the majority of the effect is intensity determining location, to allow the loop wherein intensity determines location, which is then allowed to affect intensity, would seem undesirable. The precision of the reported location (to a ten-thousandth of a pixel) would also seem questionable in these circumstances.

### Image registration issues

#### Departure from the grid

For the vast majority of beads the difference between their given and predicted locations is very small, commonly less than one pixel. However, there are often thousands of beads where the deviation is greater than a pixel, and it can commonly be as large as 4 pixels, which is a concern given the beads have a presumed radius of 2-3 pixels and between bead-centre spacings of approximately 6 pixels. Some allowance must be made for irregularities in scanning the array but, given the theoretically regular nature of the grid, this seems to be evidence that beads are being mis-registered, and there is a distinct association between the degree of departure from the grid and residual log-intensity (Additional File 7)

#### Issues of targeting the wrong bead

**Figure 3:**
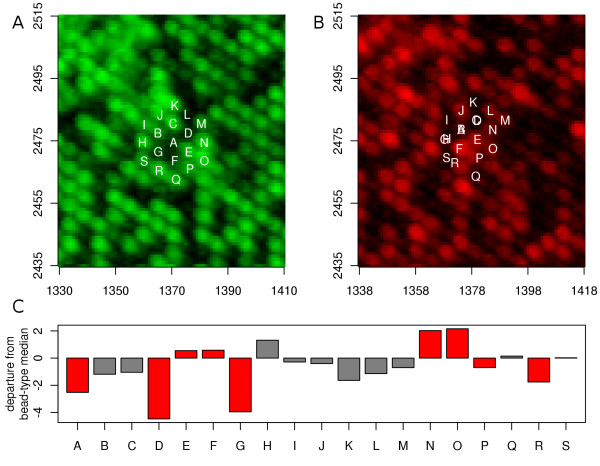
**Illustrating the failure to map bead-centres correctly in the red channel**. Illustrated are a small section of both the green (A) and red (B) images (with a slight shift to ensure alignment of the chip) from array section 4127130020_A_4. Nineteen beads are identified in the figure, and can be seen to follow a regular hexagonal lattice in the green image. The bead-centres for those nineteen beads (as provided by Illumina's software) are also plotted on the red image, where it is clear that there has been a failure in centre identification and mapping for some beads. Part C of the figure shows, for the nineteen beads, how far from the median value for the other replicates of these bead-types the reported log-intensity lies. Beads that are apparently not mapping to the correct bead in the red channel (B) are coloured red.

In such cases, the departure from the grid is so great that we must presume that the wrong bead is being interrogated. This is most apparent when considering two-colour BeadArrays. In Figure 3 we illustrate a region of a two-colour array, where a number of beads show a high departure from their predicted grid positions. Note that these could also be identified by the differences in coordinates between the two channels, or because multiple beads in the red channel share the same coordinates. 19 beads are tracked in the figure from their reported location in the green channel to their reported locations in the red channel.

Note that, of the 19 beads, A, D, E, F, G, N, O, P and R map to the wrong position in the red image. Moreover, we see three instances where distinct beads in the green image map to the same bead in the red image: (A,B), (C,D) and (G,H). The consequence of mapping to the wrong bead is clear, with many outliers being created. Bead G should map to the bright bead (in the red channel) to which bead F now maps, but instead maps to a region of low intensity. As a consequence, bead G is taken to exhibit lower intensity than is normal for that bead-type, while bead F exhibits greater intensity than does its cohort. Beads N and O should map to regions of low intensity, but instead return moderately high values as a consequence of their picking out of neighbouring beads. Naturally there are cases, such as P, where the bead-centre maps to the wrong bead in the image, but the two beads exhibit similar intensities so there is little effect.

With localized problems such as this, where frequently only a single bead in a neighbourhood shows a concerning discrepancy between the two channels, it is natural to call upon the redundancy built into the Illumina BeadArray platform and simply discard the beads in question. In these circumstances, correcting a few extra beads would be unlikely to reward adequately the considerable efforts required. The task then is one of identifying these beads for removal, about which we now add a note of caution.

#### Variable pixel size

When comparing the coordinates of beads from the two channels, one will immediately be struck by a discrepancy. Since the array is not in exactly the same position in the two images, a shift of one coordinate set will be required, often by a distance of ten or more pixels. However, closer inspection will show that this shift is not constant across the image. The nature of this shift is illustrated in Additional File 8. Two things are clear. First, the array does not occupy the same number of pixels in the red and the green channels, therefore a pixel represents a different physical size between the two channels. Second, for at least one of the channels, the physical size represented by a pixel changes along the array within the channel.

Not only does this have implications for the identification of beads that deviate from the expected grid, but also raises more fundamental questions about the use of a constant number of pixels to calculate foregrounds and backgrounds. Moreover, having seen that the fractional part of a bead's coordinates is influential on the derived intensity, we may ponder what might be the implications of such a changing shift between the two channels.

#### General mapping of images to beads

Sometimes the image registration can go dramatically wrong, resulting in the entire grid of bead-centre locations being positioned incorrectly within the image (Additional File 9). Due to Illumina's random construction method, the consequence of such mis-registration is that the beads have random annotation. The intensity values associated with each bead-type are then a random sample of intensities, and their summarization produces an estimate of the average intensity on the array.

**Figure 4:**
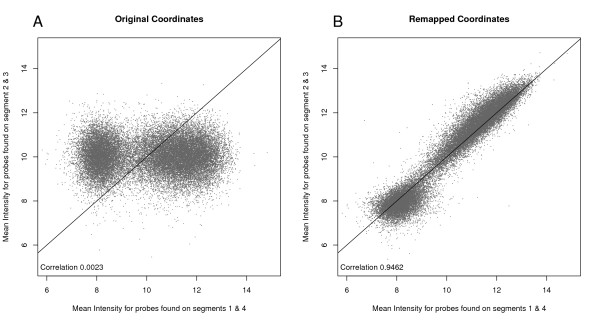
**Demonstrating the effect of remapping the probe annotation**. For the array illustrated in Additional File 9, bead intensity was averaged by bead-type across segments 2 and 3 (the mis-registered segments) and compared with the average intensity for the same bead-type from segments 1 and 4 (the successfully registered segments). The bead-type identities for segments 2 and 3 were then remapped by manually shifting the grid of bead-types to achieve alignment with the figure, but retaining the individual bead intensities that had been returned.

Figure 4A illustrates the agreement between two segments of an array that have been mis-mapped, and two that were apparently well-mapped. There is no agreement, and the range of values obtained from the mis-mapped segments is much narrower (consistent with each being an estimate of the array's average intensity). Re-mapping the bead-types demonstrates that there is still information in the array that can be unlocked by a careful bead-level analysis (Figure 4B).

We can automate the re-mapping of bead-types. When the grid of bead IDs is correctly positioned, the variance within each bead-type should be significantly lower than when they are mis-mapped. Being out by a single column or row completely scrambles the annotation, so there should be no improvement until the correct mapping is achieved. We search multiple shifts of the grid and identify when the they are correctly aligned by the drop in variance. Additional File 9 shows, for the mis-registered segment, how the within-bead-type variance drops when the two mis-mapped segments are moved 6 rows vertically. For the two correctly aligned segments, any change in the grid position increases the variance.

### Influence of local phenomena

#### Beads neighbouring non-decoded beads

We have mentioned already that the decoding process is not perfect and it is common for a small percentage of beads to fail this identification step. The locations of the non-decoded beads fall into two distinct patterns. Many non-decoded beads occur apparently randomly scattered across the array. Others however occur in compact clusters in a manner clearly not independent of one-another, and consistent with a localized technical issue that could have interfered with the decoding step.

**Figure 5:**
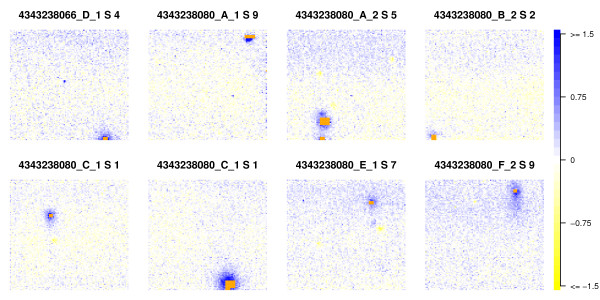
**Beads neighbouring clusters of non-decoded beads**. Illustrated are 8 segments from various sections of the expression data, each showing a cluster of non-decoded beads (orange) surrounded by a distinct region of high intensity (blue) beads. Within-bead-type residual intensities (log-intensity minus median log-intensity for that bead-type) have been averaged over beads in 20 × 20 pixel squares, and the colour scale for each segment is calculated separately (in each case going from yellow for the lowest value to blue for the highest). Clusters of non-decoding beads are a feature of array manufacture rather than processing, with the implication that the regions of high intensity are similarly so. Images are indexed in the form A_B_C S D, where A is the chip name, B the sample name, C the section number, and D the segment number.

We have observed that it is common for successfully decoded beads, in the vicinity of such clusters, to display higher intensity than is expected for their bead type (examples of which can be seen in Figure 5). Such behaviour is suggestive of a technical artefact that extends beyond the area that failed to be decoded, with the implication that these beads should be removed. Techniques to identify artefacts of this nature, such as BASH [10], will struggle to find the artefact because of the cluster of non-decoded beads in the middle. The identification of the clusters themselves though is relatively straightforward (especially if using the complete bead locations from the .*locs *file), and exclusion of beads around such clusters would be easy to implement. The influence of this bias across an experiment is illustrated in Additional File 10.

#### Beads neighbouring 'encroaching' beads

**Figure 6:**
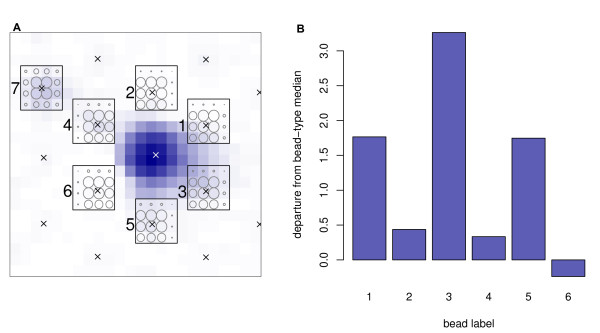
**Signal overspill from a bright bead onto its neighbours**. (A) A false-colour image of section 4343238080_B_2 showing the region surrounding a bright bead centred at pixel (377, 734). The highest intensity pixels are indicated in dark blue, whilst the dimmest pixels are white. Bead-centre locations are marked with a cross. Each of the black squares show the 16 pixels used to calculate the foreground intensity for the neighbours, with the size of the circles in each square representing the weight attributed to each pixel during the foreground calculation. Neighbours 1, 3 and 5 appear to fall largely within the signal emitted by the bright bead. Bead 7 is included as an example where the bead-centre was identified between pixels, resulting in a more even weights matrix. (B) A bar chart showing the difference between the calculated log intensity for each of the neighbouring beads and the median log intensity for beads of that type. Those beads that fell within the signal emitted by the bright bead are all seen to have a dramatically higher intensity score than is expected for their respective bead-types.

Particularly bright beads can encroach into the region used to calculate the foreground of neighbouring beads. Figure 6 shows one such example, where several beads fall within the region of fluorescence resulting from a bright neighbour. In particular, the regions used to calculate the foreground intensity of beads 1, 3 and 5 appear to overlap with the signal emitted from the bead in the centre.

Naturally, we must be wary of the possibility that the central bead is not encroaching, but that the satellite beads are showing a signal and that we are simply unable to observed the boundary between them. This may be the case for bead 4, but there are two reasons for believing this not to be the case for the other beads. Firstly, we would expect a local mode of intensity at the satellite bead if this were the case (as there is with bead 4), and secondly we would expect the satellite beads to show similar intensities to beads of a similar type elsewhere on the array.

Figure 6 also shows the difference between the foreground intensity of each of the neighbouring beads and the median intensity for beads of the same type. It is clear that for three of the neighbouring beads (the three over which the fluorescence from the central bead is most visibly encroaching) the foreground intensity is considerably higher than the average for that bead type. Illumina's sharpening step, performed before the foreground intensities are calculated, should go some way to accounting for such encroachment. Given the high residual values for the beads neighbouring a bright bead in Figure 6, which were calculated following this sharpening, it appears that the sharpening may not be adequate for such a purpose (but may still be desirable) [8]. A simple remedy would be to down-weight (or entirely remove) beads that have particularly bright neighbours when summarizing Illumina data. The influence of this bias across an experiment is illustrated in Additional File 10.

#### Beads with aberrant background calculations

Out of the 289 pixels that are considered for the background calculation, the number that are "true background" pixels for a given bead ranges from 117 to 237 for one expression array (4343238066_A_1), with median 149. Note that if we took a 6 × 6 square foreground mask for each bead, more than two-thirds would be considered to have no "true background" pixels within their 289 candidates. However since the outer layer of that 6 × 6 square makes a negative contribution to the foreground calculation, it is reasonable to use the 4 × 4 pixel mask. As anticipated, when there are more true background candidate pixels, the non-robust measure of background tends towards more extreme values. The effect is not large in magnitude however, due to the generally low variability in background values, and so is unlikely to be a concern (see Additional File 11).

**Figure 7:**
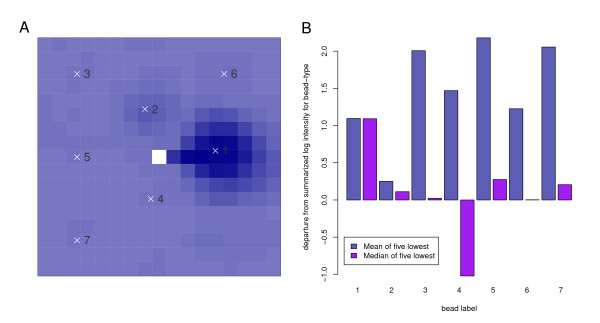
**Pixels of unusually low intensity and their influence**. (A) Illustrating the occurrence of an unusually low intensity pixel. A region, centred on pixel (964, 6081), from image 4343238066_A_2_Grn.tif, is illustrated. Whilst the majority of the image shows the typical background log-intensity, a single pixel with an incredibly low value is observed. Note that this occurs in the vicinity of an almost-saturated high-intensity bead. Seven beads which include this pixel in their background region have been successfully decoded, and their bead-centres are indicated. (B) Illustrating how the intensities of the affected beads show a large deviation from the summarized value for their respective bead-types. If the median of the five lowest pixels is used instead of the mean during the background calculation, the impact of the low intensity pixel is reduced in most cases.

It is not uncommon to see the occasional pixel, in an Illumina image, that has an intensity much lower than the general background pixels. Such pixels often occur in proximity to extremely bright pixels (see Figure 7A), suggesting that this is an artefact introduced by the image capturing device.

When such pixels fall within the background calculation region for a bead, they inevitably end up as one of the five lowest pixels in the region and so contribute to the background score for that bead. The resultant background scores for such beads are lower than they would otherwise be, and the final intensity for the bead is calculated to be higher than it should. There are few such pixels on an array, and their identification and exclusion from calculations would not present a technical challenge, however a simple change to the background calculating rule might present the simplest solution. Currently the mean of the five lowest pixels is used, however using a trimmed mean of a certain number of the lowest pixels or simply using the pixel of a certain rank (equivalent to the median of the *n *lowest pixels for some value of *n*) would be alternatives to consider. Figure 7B shows that altering the background calculation to use the median of the five lowest pixels, rather than the mean, decreases the departure from the bead-type type median for the beads in close proximity to the pixel shown in Figure 7A. The influence of this bias across an experiment is illustrated in Additional File 10.

#### Beads neighbouring similar beads

In investigating whether neighbouring beads of the same type can affect the physical chemistry of hybridization to an extent that the registered intensity will also be affected, we find a median of 150 pairs of neighbouring twins (IQR: 142:5 to 159:5) across the 24 sections of the two expression chips. We can detect no significant association between the presence of a neighbour of the same bead-type and observed intensity (Additional File 10), but it should be noted that the range of intensities over which such an effect might be strongest is also that associated with low signal to noise, so we might anticipate having low power to investigate such a phenomenon.

The observed numbers of pairs of such beads far exceed the numbers we expected from theory, or based on a permutation test (Additional File 12). Only two explanations seem plausible for the excessive number of neighbouring twins that we see in the real data. Either the beads are not independently arranged on the array, or the beads are being decoded incorrectly in a manner that leaves them with their neighbour's identity. Should either the physical chemistry (following target depletion) or the various possibilities for explaining the excess neighbour pairs raise concerns, then identification of such bead-pairs is a straightforward task given that the identification of the complete network of neighbouring beads is already a necessary task for various bead-level preprocessing steps. Identification of beads that neighbour similar beads would fall out of this preparation and allow for their down-weighting or exclusion.

### Impact on biological interpretation

**Figure 8:**
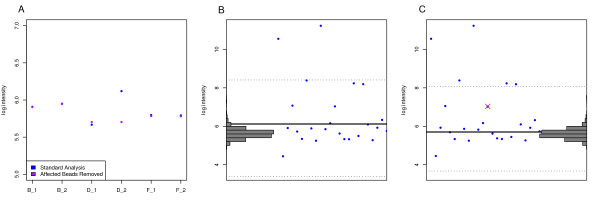
**Effect on biological interpretation**. (A) Showing the log intensity of bead-type 5900598 from six array sections each of which had the same sample hybridized to them. The summarized intensities have been calculated twice, once using the standard analysis and once with beads affected by the identified phenomena removed. (B) & (C) show the log intensties of the individual beads of type 5900598 on section 4343238080_D_2 calculated using the standard and two-step summarization methods respectively. Histograms of the log intensities of the negative control beads calculated in the same fashion are shown down the sides. In panel (C) beads excluded due to their proximity to the phenomena identified in this study are indicated by the red cross. The dotted lines indicate the range of values outside of which beads are classed as outliers and are excluded from the summarization step. The removal of the marked bead results in three additional beads being classed as outliers. The result is a lower summarized intensity (the solid black line), which when compared to the negative control beads, changes from being classed as expressed to not expressed.

Figure 8 gives an example of how masking affected beads prior to summarization offers an improvement over the standard outlier removal process. Taking the 6 sections from chip 4343238080 to which the MAQC Universal sample was hybridized, the first panel (A) shows summarized log intensity for bead-type 5900598 using both the standard summarization and one following the removal of affected beads. We can clearly see that the correspondence between the two replicate sections D1 and D2 improves markedly following this two-step summarization. Panels (B) and (C) show the log intensities for the individual beads of this type, using the standard and modified analyses respectively, along with the histograms of negative control beads, which are used to call whether a bead is being expressed.

In this example the masked bead would normally be included in the summarization. However, its removal results in three additional beads being classed as outliers and excluded from the summarization. This in turn dramatically lowers the summarized intensity and results in the bead type being classed as not expressed, a finding that is corroborated by the replicate sections.

## Conclusions

Some of the arrays we present here feature the mis-registration of beads on a scale that can have only one of three outcomes. Either the array will be omitted from analysis (at the expense of a replacement), it will be included 'as is' (but be detrimental to data quality), or can be included after correction (such as the approach we present here). The remaining phenomena we have described will have a lesser impact, but occur apparently more frequently (summarized in Table 1), and, while not ruining an experiment, will impact upon data quality.

**Table 1:** Number of affected beads for expression arrays.

Section ID	Neighbouring bright bead	Large deviation from grid	Near abnormally low pixel	Neighbouring non-decoded cluster
66_A_1	54	501	32	13059
66_A_2	30	589	32	4312
66_B_1	210	373	33	1062
66_B_2	140	340	30	5511
66_C_1	12	443	14	1705
66_C_2	12	514	8	709
66_D_1	72	392	8	272
66_D_2	60	382	4	2616
66_E_1	6	495	7	1918
66_E_2	24	508	0	9399
66_F_1	108	625	25	4427
66_F_2	54	475	12	4800
80_A_1	66	451	38	1579
80_A_2	42	343	48	2530
80_B_1	420	348	44	212
80_B_2	342	360	76	341
80_C_1	54	281	40	2035
80_C_2	30	457	50	0
80_D_1	15246	5431	148	0
80_D_2	12864	5653	71	495
80_E_1	162	719	43	2058
80_E_2	6	506	13	568
80_F_1	30	341	52	381
80_F_2	78	355	28	880

Median	57	454	32	1642

If beads are not providing sound estimates of the quantity that they are designed to measure, yet by chance return an intensity consistent with the true target, then they will have the effect of increasing the perceived number of beads contributing to the estimate and thus will lead to artificially low standard errors and consequently to erroneous results. Since low standard errors are inherently desirable, it is unlikely that concerns will be raised in such cases.

When the intensity is inconsistent with the true target, there is a chance that the standard Illumina analysis will identify these beads as outliers. However it is sub-optimal to use such a broad tool for this purpose, when more targeted options are available. Moreover, we have shown an example where the effect of a miscreant bead is to obscure the outlying status of other beads for which we have no explanation of their abberant intensities. These are the beads for which a general outlier removal step is warranted, and we have shown that a two-pass approach of removing known problems and then applying a general outlier removal can be beneficial.

We summarize our findings in Table 2, where we list the problems identified, with associated diagnostics, solutions, and details of where the solutions are implemented. Most alarming is that the registration of images produced by Illumina scanners can be subject to several forms of error, which could greatly impact upon the downstream results drawn from these images. The mis-registration can take at least two forms, the most obvious of which is the mis-alignment of an entire segment's bead-centres. More subtle, small, mis-registrations can result in local bead-centres missing beads or two bead-centres being assigned to the same feature in a image whilst other features are ignored.

**Table 2:** Summary of results.

Problem	Diagnostic	Solution	Implementation (where implemented)
There is local discordance between the locations in the two channels from two-colour arrays	Between-channel differences in location can be compared to local median differences	If one channel is clearly wrong (from relative grid positions in that channel) then the bead-centre can be remapped from the other channel, else the bead should be dropped	

Image cropping out part of the array section, so that values cannot be calculated	Bead-centre coordinates lie without the dimensions of an image, beads apparent on edge of image	Exclude beads with such coordinates	Any text editor can assess the coordinates, while *beadarray *contains code for the reading and plotting of images on a useful scale. *beadarray *allows for the masking of beads to be excluded in analyses.

Beads are mostly well-registered, but grid of bead-centres does not align with the image resulting in scrambling of bead-type data	Visual inspection of bead-centres over image. Without access to images, check that the segments have similar extreme x coordinates and that they are equally spaced along the y-axis	In extreme circumstances, bead IDs can be remapped, but usually segments/sections should be excluded	*beadarray *contains code for the reading and plotting of images, as well as the over-laying of bead-centres. *beadarray *allows for the masking of beads to be excluded in analyses. Scripts are provided for automatic re-mapping

Neighbouring beads of the same bead-type are a potential concern (albeit unsubstantiated)		Such pairs of beads can be identified and down-weighted or excluded	*beadarray *contains code for identifying the network neighbourhood. *beadarray *allows for the masking of beads to be excluded in analyses.

Beads neighbouring clusters of non-decoded beads are more likely to take extreme values	The presence of such clusters can be determined from the .*locs *file	Exclude/Down-weight beads in a zone about such clusters	*beadarray *allows for the masking of beads to be excluded in analyses.

Bright beads encroach on neighbours, raising their associated values	Visual inspection of the brightest beads	Bright beads can be identified (by intensity or size using EBImage) and their neighbours down-weighted or excluded	*beadarray *contains code for the reading and plotting of images, as well as code for identifying neighbours. *beadarray *allows for the masking of beads to be excluded in analyses.

Abnormally low pixels in the image distort background values and so final intensities	Pixels present with values noticeably lower than the mode	Exclude such pixels, or use a less-sensitive background calculation rule	*beadarray *contains code to read images, which allow for tests of the distribution of intensities within R. *beadarray *allows alternative background calculation rules

Multiple bead-centres map to the same location	Text files (or .*locs *files) can be scanned for neighbours that are unusually close	In two-colour arrays, it may be clear which is the correct bead-centre, else exclude both	Scripts provided to detect departure from predicted bead-centre.

Summarizing the phenomena we have described, methods for their diagnosis, and possible solutions.

In addition to this, we have demonstrated that in theory the relationship between the position of a bead on the array and the grid of pixels introduced by creating a digital image of the array during scanning can affect the intensity value attributed to that bead. It appears that the majority of the association seen between bead-intensity and within-pixel bead location can be attributed to the intensity of a bead affecting the location at which it is found, yet for a bead to lie away from its anticipated location on the grid we have shown to be indicative of a biased intensity. It may be that different approaches for identifying beads and computing intensities could reduce or eliminate this source of variation.

Finally we have shown that there are a variety of spatial effects that may impact on the reliability of results for individual beads. Particularly bright beads appear to display a 'bleed over' effect in which neighbouring beads are swamped by the signal being emitted from the bright bead. We have also identified isolated pixels with intensities far lower than the modal background for the array. Each of these artificially inflates the intensity attributed to their neighbouring beads, an effect also seen in the neighbourhood of clusters of non-decoded beads.

Yet if they can be identified as suggested in the results and in Table 2, beads can easily be down-weighted or excluded from an analysis. The degree of replication on Illumina arrays often allows for beads to be excluded while still making estimation possible. Unfortunately it is still the case that, on some occasions, entire arrays must be forsaken, so the need for robust experimental design is not negated by these salvage techniques. We have also noted evidence of a lack of independence in the random layout of the BeadArrays. This in itself we have not shown to bias results, however it does suggest that one can not simply trust in an IID random layout of beads to overcome biases introduced by spatial artefacts.

The identification and remedy of the problems we have described are reliant upon access to the raw, bead-level, data. Even if one plans to analyse summarized data using Illumina's software, the routine recording of bead-level data provides a safety net should problems with the arrays be identified or suspected. With such routine access to bead-level data, an automated pipeline for quality assessment could be implemented painlessly, allowing the detection of the artefacts described here, and would provide reassurance regarding an independent summary-level analysis for experimenter, reader, and reviewer alike. Given this we highly recommend the collection and storage of bead-level data.

## Authors' contributions

MLS conducted analyses, authored code and drafted the manuscript. MJD provided Illumina expertise and helped to draft the manuscript. ST provided statistical expertise and helped to draft the manuscript. AGL conceived the study, provided statistical expertise, conducted analyses and drafted the manuscript. All authors read and approved the manuscript.

## Supplementary Material

Additional file 1 R script containing functions that are not available in beadarray Click here for file

Additional file 2 **Vignette to reproduce figures and tables in this paper**. Click here for file

Additional file 3 Figure illustrating the Illumina foreground calculation. Click here for file

Additional file 4 Figure demonstrating the between-bead dependence of background calculations. Click here for file

Additional file 5 Flowchart illustrating preprocessing steps. Click here for file

Additional file 6 Figure illustrating the results of the bead simulation exercise. Click here for file

Additional file 7 Figure illustrating the association between departure from the grid and intensity. Click here for file

Additional file 8 Figure illustrating the inconsistent shift required to align red and green images of the same section. Click here for file

Additional file 9 Figure illustrating an example of image mis-registration. Click here for file

Additional file 10 Figure illustrating the influence of various biases when considered across an entire experiment. Click here for file

Additional file 11 Figure illustrating the influence of the number of true background pixels that a bead has on its calculated background. Click here for file

Additional file 12 The numbers of neighbouring pairs of beads of the same type, observed and theoretical values. Click here for file
