# Supplementary material for: Identification and correction of previously unreported spatial phenomena using raw Illumina BeadArray data
Source: BMC Bioinformatics. 2010 Apr 27;11:208. doi: 10.1186/1471-2105-11-208 (PMC2880029; doi:10.1186/1471-2105-11-208)
Supplement: Additional file 3 — Figure illustrating the Illumina foreground calculation. [file 1471-2105-11-208-S3.PDF]

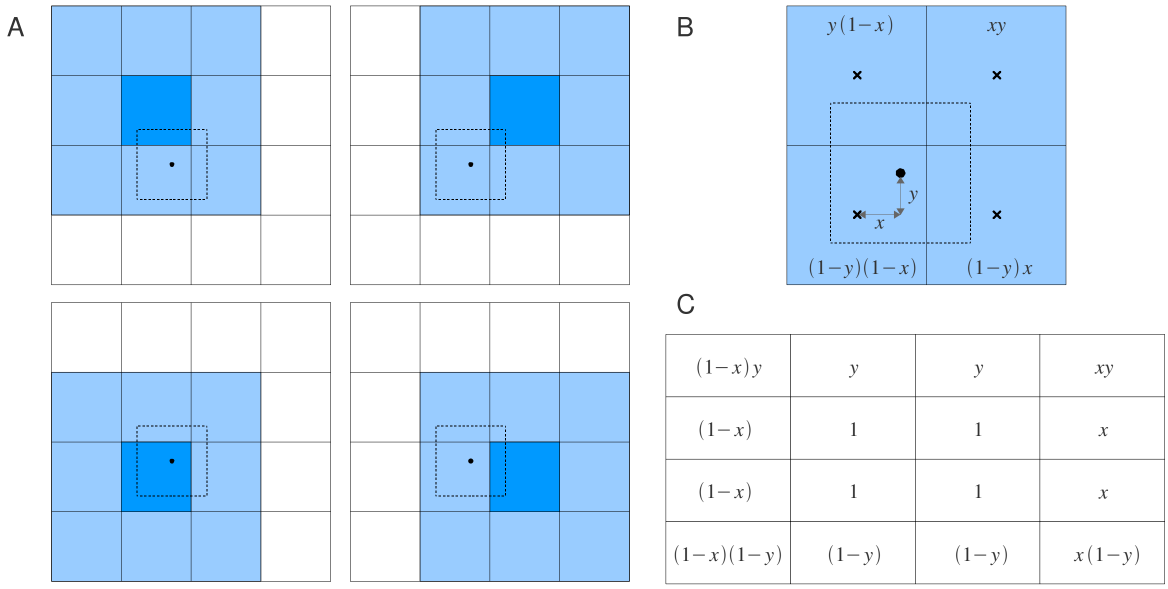

$(1-x)y$	y	y	xy
$(1-x)$	1	1	x
$(1-x)$	1	1	x
$(1-x)(1-y)$	$(1-y)$	$(1-y)$	$x(1-y)$

(A) Illustrating how a virtual pixel is placed over a bead centre, defining the four pixels in the image that lie closest to the bead-centre. For each of these four pixels, intensities are averaged over a 3×3 pixel square (illustrated) and a weighted average of the four subsequent values is returned. (B) The weights are determined by the fraction of the virtual pixel that overlaps the real pixel, which (if the fractional parts of the bead centre coordinates are denoted x and y) will always come to be xy, $x(1-y)$, $y(1-x)$ and $(1-x)(1-y)$ in some order. Note that the fractional part of the coordinate is zero at the centre of a pixel. (C) Naturally, this process can be represented as a 4×4 matrix of weights, and this matrix is depicted. Note that the four central pixels receive equal and maximum weighting regardless of the values of x and y.
