# Supplementary material for: Identification and correction of previously unreported spatial phenomena using raw Illumina BeadArray data
Source: BMC Bioinformatics. 2010 Apr 27;11:208. doi: 10.1186/1471-2105-11-208 (PMC2880029; doi:10.1186/1471-2105-11-208)
Supplement: Additional file 4 — Figure demonstrating the between-bead dependence of background calculations. [file 1471-2105-11-208-S4.PDF]

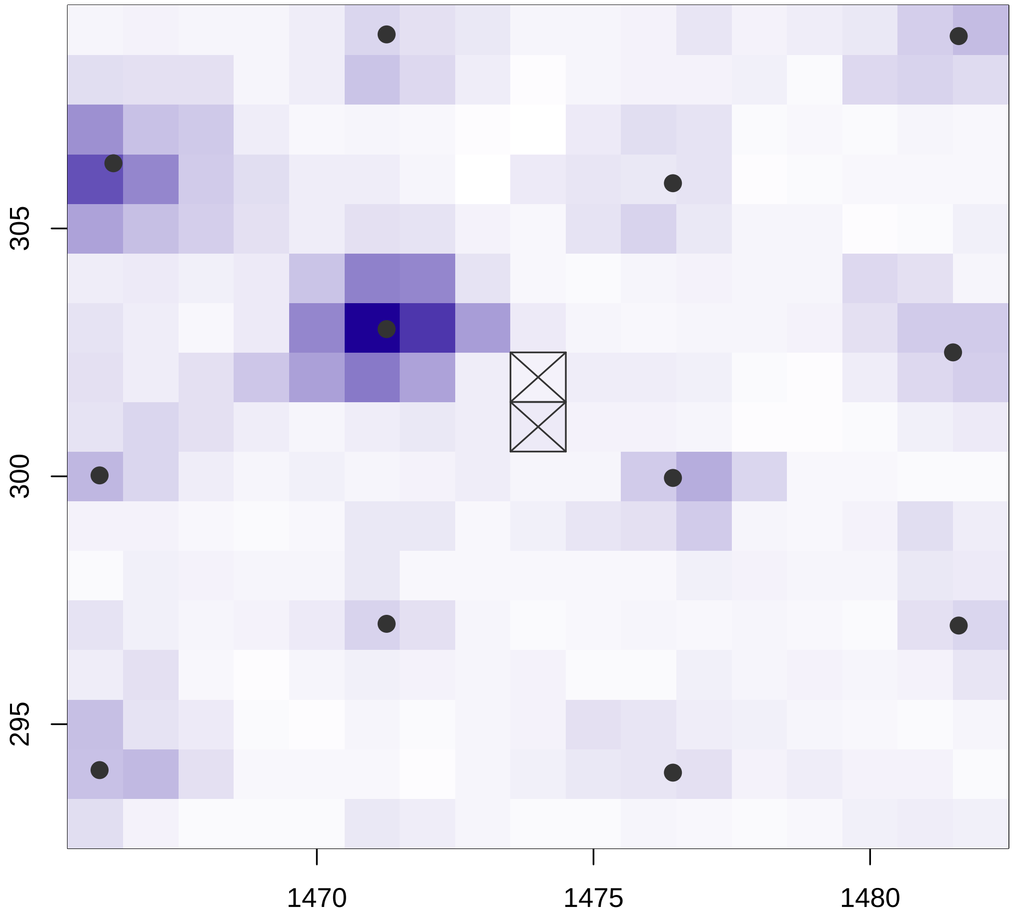

Illustrated is a 17×17 pixel square containing 12 bead centres. By definition, the central pixel will be within the background calculation area for all 12 of these beads (as indeed will one of its neighbours - both indicated in the figure). Should one or both of these common pixels exhibit an aberrantly low intensity, then the intensities associated with the 12 pictured beads will be biased in a correlated manner.
