# Supplementary material for: Identification and correction of previously unreported spatial phenomena using raw Illumina BeadArray data
Source: BMC Bioinformatics. 2010 Apr 27;11:208. doi: 10.1186/1471-2105-11-208 (PMC2880029; doi:10.1186/1471-2105-11-208)
Supplement: Additional file 5 — Flowchart illustrating preprocessing steps. [file 1471-2105-11-208-S5.PDF]

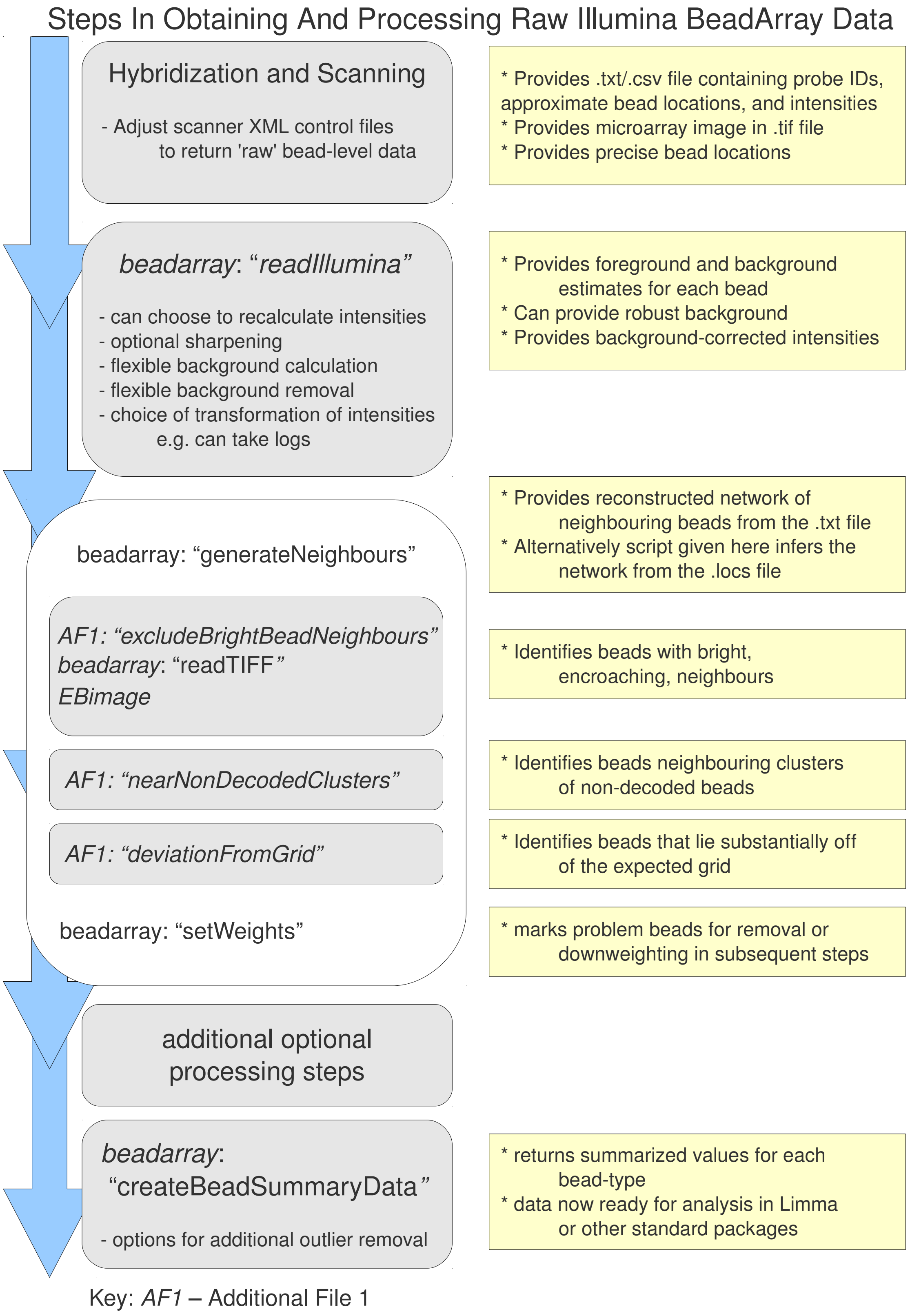

Flow chart detailing the steps utilised to obtain and process raw Illumina BeadArray data. Package and function names are given in the grey boxes, along with descriptions of each step.
