# Supplementary material for: Identification and correction of previously unreported spatial phenomena using raw Illumina BeadArray data
Source: BMC Bioinformatics. 2010 Apr 27;11:208. doi: 10.1186/1471-2105-11-208 (PMC2880029; doi:10.1186/1471-2105-11-208)
Supplement: Additional file 6 — Figure illustrating the results of the bead simulation exercise. [file 1471-2105-11-208-S6.PDF]

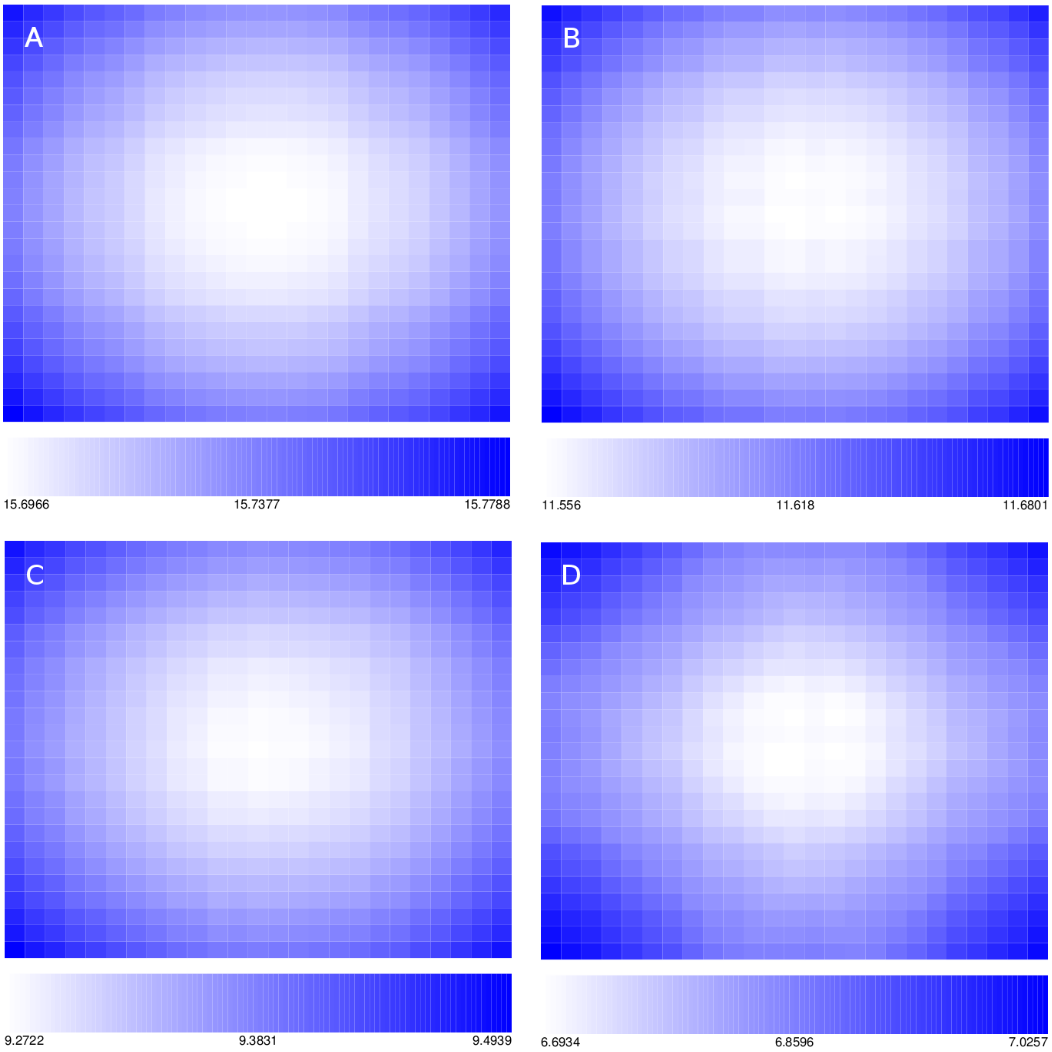

Each panel shows how the calculated foreground intensity changes as a bead moves relative to the pixel lattice. In each case a bead was created with the fractional parts of its centre coordinates set to 0. The bead was then pixelated and the resultant foreground intensity calculated. The bead was then moved in increments of 0.04 pixels in both the X & Y directions and the process repeated.

In order to assess the impact on beads of differing brightness each panel represents a bead of differing size and intensity. From (A) to (D) the simulated beads had diameters of 6, 5, 4 & 3 pixels and maximum $\log_2$ intensities at the bead centre of 16, 12, 10 & 8 respectively.
