# Supplementary material for: Identification and correction of previously unreported spatial phenomena using raw Illumina BeadArray data
Source: BMC Bioinformatics. 2010 Apr 27;11:208. doi: 10.1186/1471-2105-11-208 (PMC2880029; doi:10.1186/1471-2105-11-208)
Supplement: Additional file 7 — Figure illustrating the association between departure from the grid and intensity. [file 1471-2105-11-208-S7.PDF]

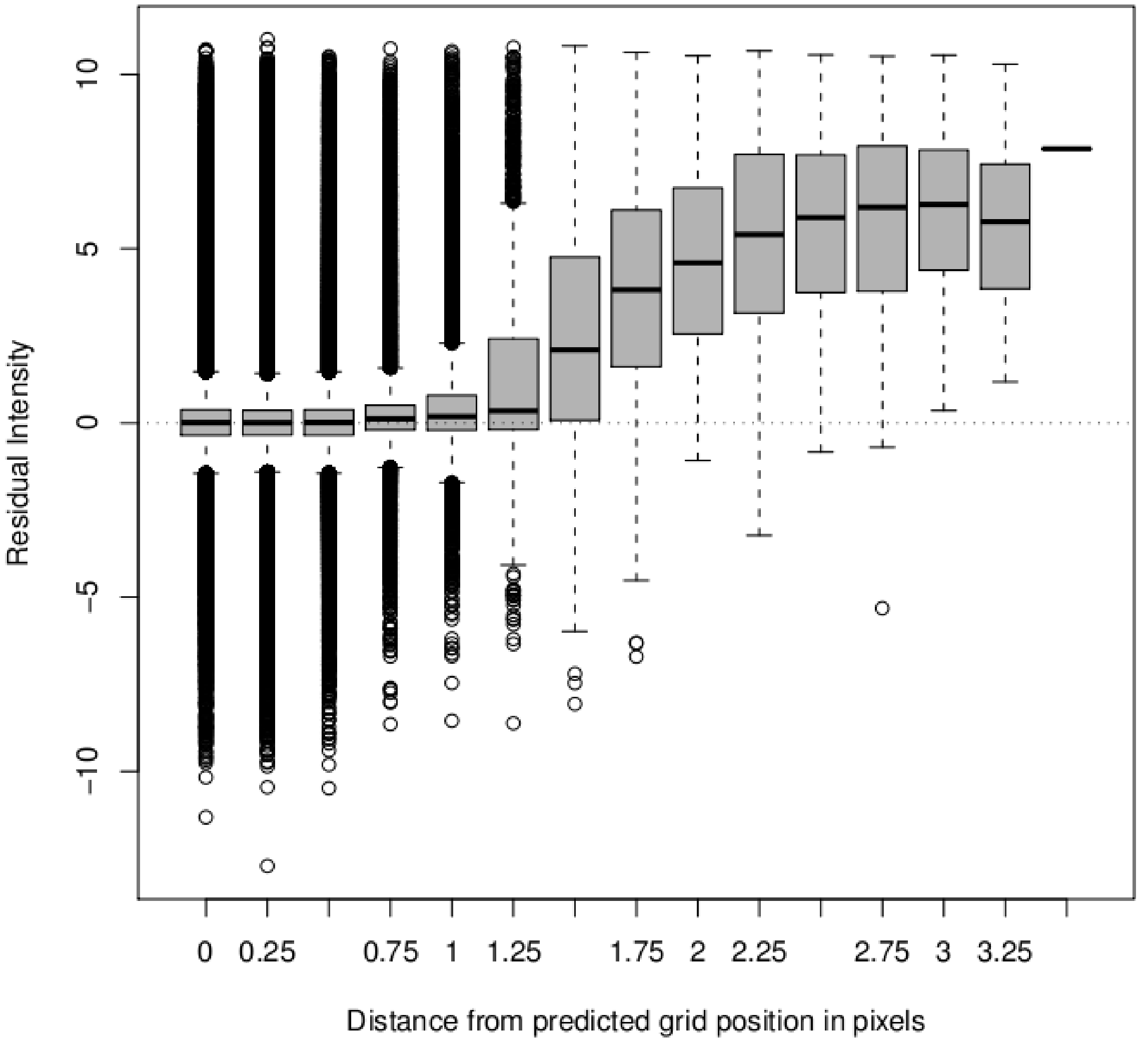

Illustrated is the association between deviation from predicted grid location and residual log intensity for array section 4343238080_D_1. Using the complete list of bead centres, taken from the *.locs* files, each of the nine segments was considered in isolation. A linear model was fitted to grid of bead centres and the resulting coefficients were then used to estimate the ideal location of each the bead centre. The euclidean distance (in pixels) was then calculated between the actual and predicted positions for the whole section.
