# Supplementary material for: Identification and correction of previously unreported spatial phenomena using raw Illumina BeadArray data
Source: BMC Bioinformatics. 2010 Apr 27;11:208. doi: 10.1186/1471-2105-11-208 (PMC2880029; doi:10.1186/1471-2105-11-208)
Supplement: Additional file 8 — Figure illustrating the inconsistent shift required to align red and green images of the same section. [file 1471-2105-11-208-S8.PDF]

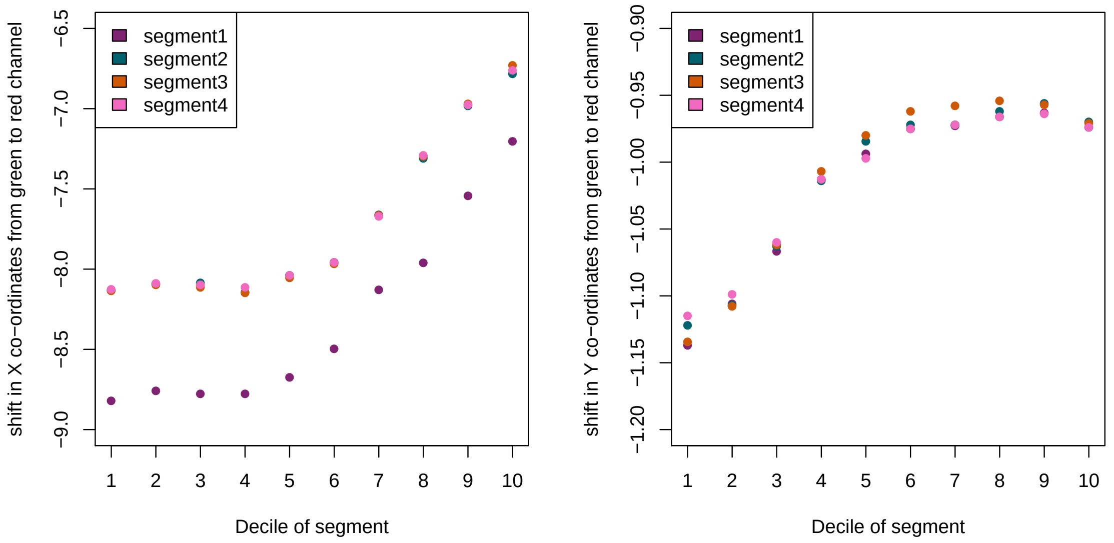

Depicted are the coordinate shifts from the red image to the green image required to align beads across the two channels for array section 4127130188_B_9. The section in question divides naturally along the Y-axis into 4 segments, and then along the X-axis has been divided into deciles. Each of the resulting 40 regions contains $> 10,000$ beads. Note that the X coordinate shift shows greater variation that that of the Y coordinate, and that generally greater variation is seen along segments than between them.

Taking one CNV370-duo section, we divided each of the four segments into 10 parts and calculated the median X and Y coordinate shifts (according to Illumina's reported coordinates for the two channels; X relates to distance along a segment and Y to distance along the longer side of a section - note that this is contrary to intuition in the orientation that is usually considered) for beads in the resulting 40 regions. Here, region membership is defined by the green channel coordinates. Figure 10 shows these median shifts. It is unusual for a section to demonstrate such a change between segments, but the trend along a segment is typical of other arrays. Given that we seek to identify bead centres that differ by as little as two pixels after the images have been aligned, it is clear that these systematic changes occur on a scale that will interfere with this task.

Since a pan-section adjustment is not appropriate, and a bead-by-bead adjustment would be too computationally expensive for most purposes, we suggest two feasible approaches to this problem. The first is the simple approach we have used here, wherein one subdivides the section into smaller units and treats these as homogeneous blocks. There will of course be edge effects in these blocks, but if one can satisfy the tension between using small enough units that these edge effects are so small as to not matter, yet large enough that the computational load is not too great, then this need not be a concern. The alternative, which is more appealing although computationally more expensive than the simple approach, is to sample beads across the surface of the section and then interpolate the shifts for the rest of the beads, assuming the function to be smooth.

It is worth noting that even if the shift between the two images is constant across the array section, then unless that shift happens to be an integer number of pixels (highly unlikely) the fractional parts of the coordinates in the two images will differ, with consequences for calculating the intensities as was discussed earlier.
