# Supplementary material for: Identification and correction of previously unreported spatial phenomena using raw Illumina BeadArray data
Source: BMC Bioinformatics. 2010 Apr 27;11:208. doi: 10.1186/1471-2105-11-208 (PMC2880029; doi:10.1186/1471-2105-11-208)
Supplement: Additional file 9 — Figure illustrating an example of image mis-registration. [file 1471-2105-11-208-S9.PDF]

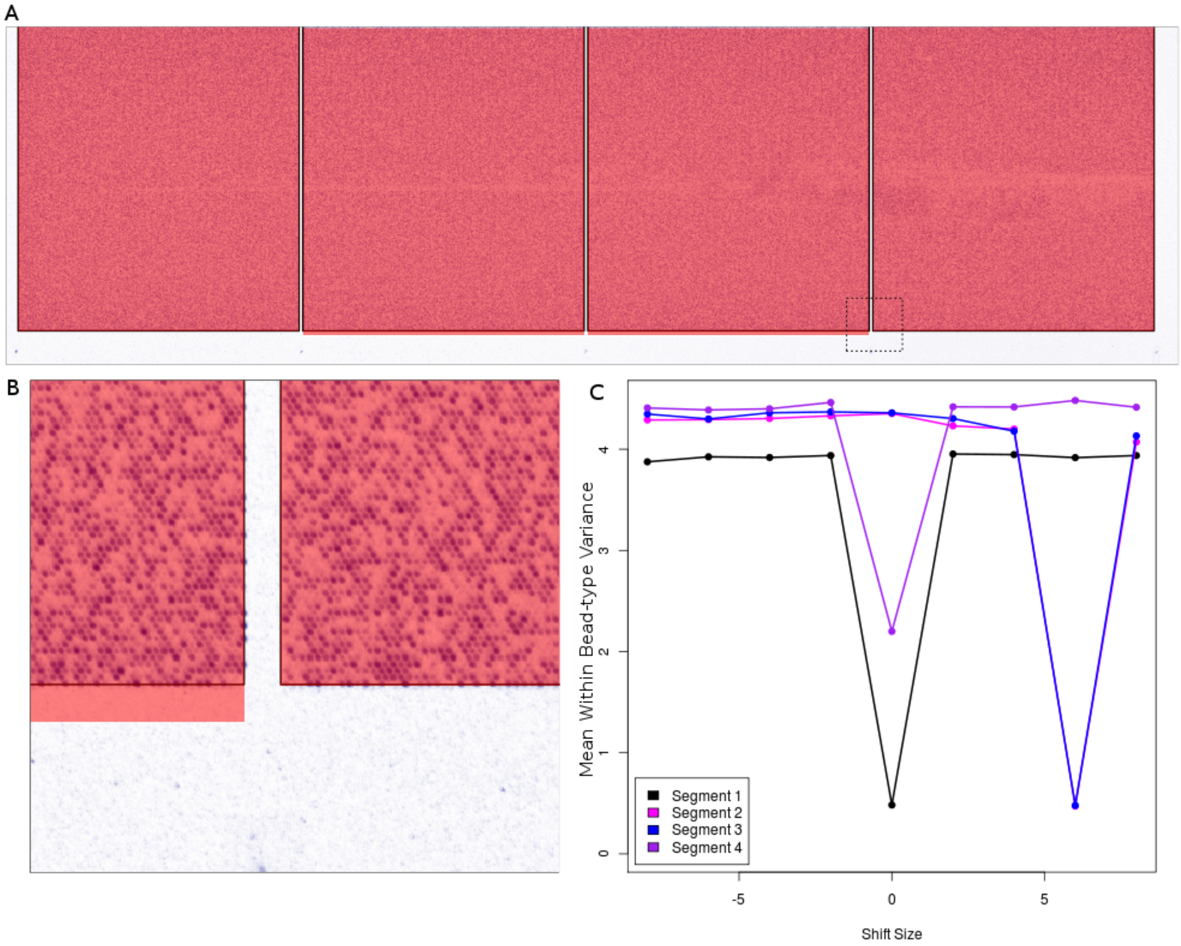

The upper image (A) is of a segment from a CNV370-duo array (4127130020_B_8). The original image has been read into R and redrawn in false colour with the pixel intensities on the log scale, making it easier to see the majority of beads in the image. The segments of bead centres have been overlaid in red. We can see that the bead centres for the middle two segments appear to have out of alignment with the true locations of the beads. Panel (B) shows a zoomed in portion of the same array (indicated by the dashed box in panel (A)). We can clearly see that the bead centres are out of alignment with their true positions for one of these blocks.

It should be noted that the array in this image is very close to the top edge, and may have been slightly cropped, which is probably the reason for the mis-registration. However, one of the benefits of using the BeadArray platform is that its degrees of redundancy often allow data to be retrieved from some sections of the array even when others have been affected by experimental artefacts. In this case the mis-registration severely hinders the recovery of any useful data.

One might expect arrays where this has occurred to be rejected by quality control procedures before any data analysis is performed. In particular, the genotype call rate may be expected to drop. However, in those arrays where we have observed this mis-registration the genotype call rate has remained above 90%. If one could identify such arrays, then under most circumstances one would exclude the array or the appropriate sections/segments where the registration has failed to such a degree. .

Panel (C) shows how the mean within bead-type variance for each segment changes as it's vertical position is altered. Each segment was shifted eight rows in each direction and the variance for each bead type was calculated. For segments 1 & 4 it is clear that no adjustment is necessary to the grid. However, both segments 2 & 3 show a clear improvement when their bead centres are moved 6 rows up, which agrees with what we can observe visually in panel (B).
