# Supplementary material for: Identification and correction of previously unreported spatial phenomena using raw Illumina BeadArray data
Source: BMC Bioinformatics. 2010 Apr 27;11:208. doi: 10.1186/1471-2105-11-208 (PMC2880029; doi:10.1186/1471-2105-11-208)
Supplement: Additional file 10 — Figure illustrating the influence of various biases when considered across an entire experiment. [file 1471-2105-11-208-S10.PDF]

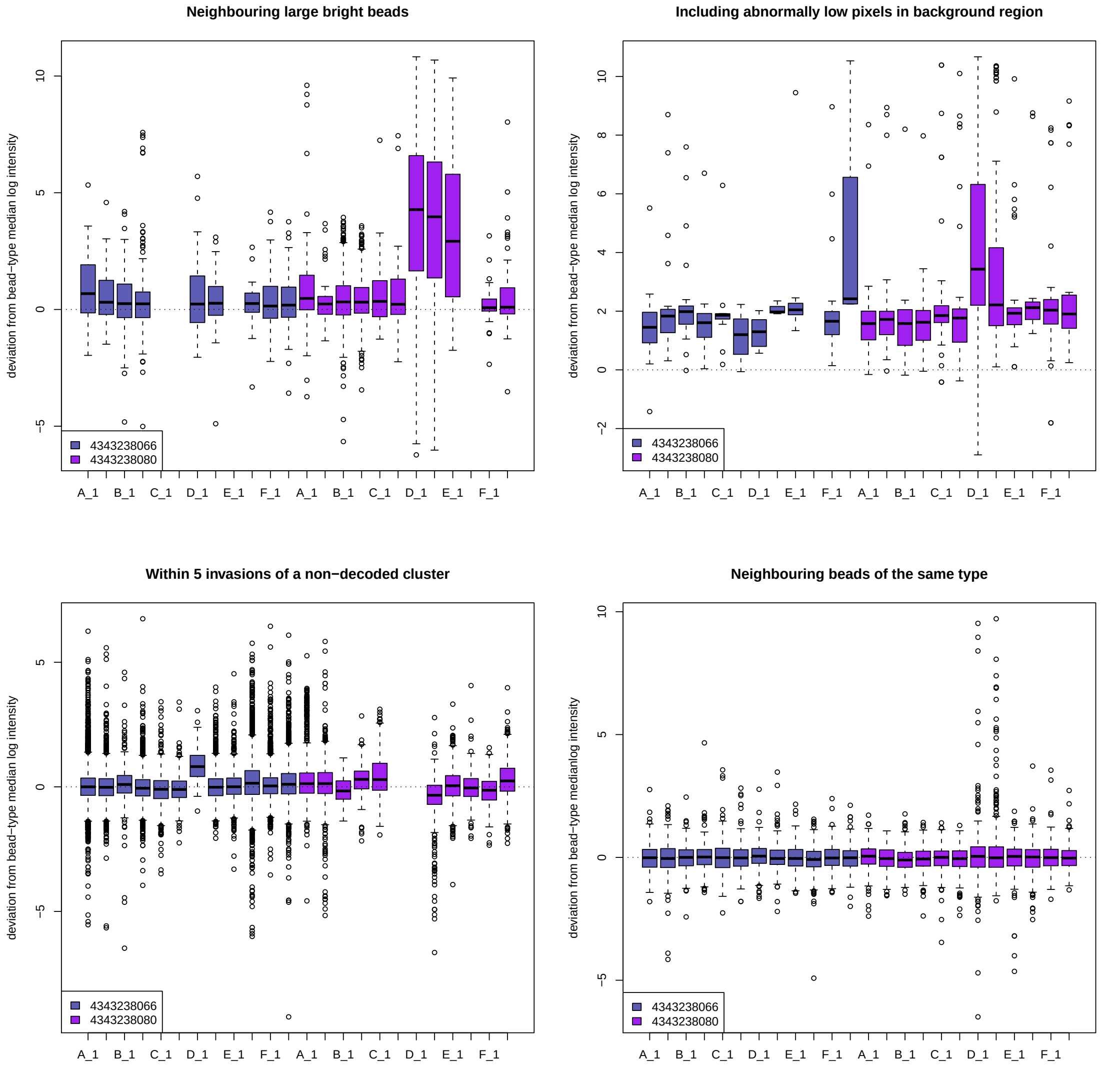

Four panels. We have shown that the biases exist with specific local examples. Here we show their influence on a larger scale. Each section from the two expression arrays was processed four times, each time identifying beads affected by a different bias. The log intensities for such beads were then compared to the median log intensity for the remaining beads of the same type on that array.
