# Supplementary material for: Identification and correction of previously unreported spatial phenomena using raw Illumina BeadArray data
Source: BMC Bioinformatics. 2010 Apr 27;11:208. doi: 10.1186/1471-2105-11-208 (PMC2880029; doi:10.1186/1471-2105-11-208)
Supplement: Additional file 11 — Figure illustrating the influence of the number of true background pixels that a bead has on its calculated background. [file 1471-2105-11-208-S11.PDF]

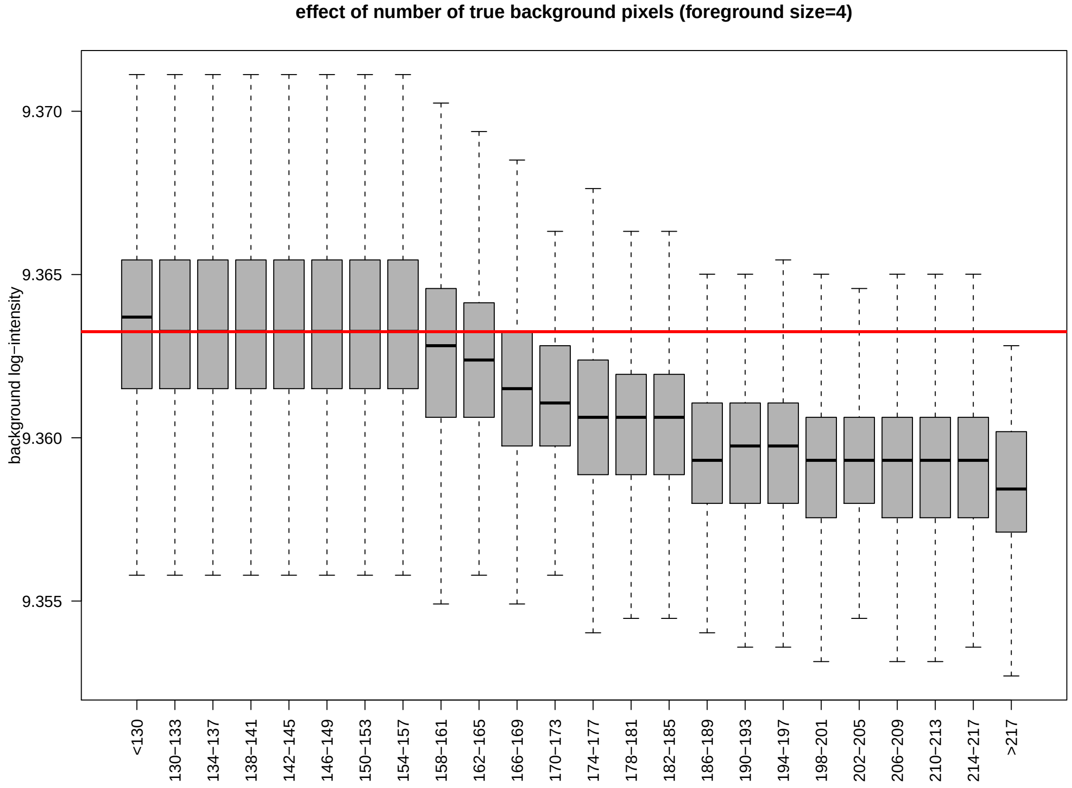

A boxplot showing the calculated background plotted against the number of “true background” pixels that were available for the calculation out of the 289 considered. The array illustrated is the expression array 4343238066_A_1, and 29 beads are omitted since they fall near an aberrant pixel such as that illustrated in Figure 7.
