# Supplementary material for: Identification and correction of previously unreported spatial phenomena using raw Illumina BeadArray data
Source: BMC Bioinformatics. 2010 Apr 27;11:208. doi: 10.1186/1471-2105-11-208 (PMC2880029; doi:10.1186/1471-2105-11-208)
Supplement: Additional file 12 — The numbers of neighbouring pairs of beads of the same type, observed and theoretical values. [file 1471-2105-11-208-S12.PDF]

Array ID	Permutation P1	P2	P3	P4	P5	P6	P7	P8	P9	P10	Observed
66_A_1	57	60	62	58	50	70	56	69	58	66	143
66_A_2	64	58	57	44	61	52	64	55	69	56	148
66_B_1	76	62	64	61	59	64	49	52	69	47	188
66_B_2	48	76	46	51	46	68	55	57	66	48	151
66_C_1	48	64	60	48	53	68	57	52	68	53	159
66_C_2	51	50	50	52	78	55	56	52	60	57	153
66_D_1	49	61	64	49	60	51	58	58	49	52	155
66_D_2	57	61	62	58	59	56	61	56	60	64	145
66_E_1	47	67	62	68	74	62	52	60	60	60	145
66_E_2	64	61	67	55	64	57	79	66	45	49	123
66_F_1	54	56	54	48	55	49	60	53	57	63	141
66_F_2	64	59	54	64	59	63	60	52	73	53	164
80_A_1	60	53	60	65	54	61	49	68	50	51	168
80_A_2	68	68	54	52	49	64	65	46	52	69	124
80_B_1	71	54	64	63	64	71	50	61	59	62	159
80_B_2	60	65	49	65	60	67	52	45	53	60	147
80_C_1	63	58	58	70	62	62	57	54	49	60	149
80_C_2	54	59	70	40	65	70	73	58	62	53	158
80_D_1	56	52	71	58	51	51	56	58	62	68	142
80_D_2	52	54	70	52	66	60	64	64	63	66	162
80_E_1	46	66	56	56	52	54	72	71	48	56	118
80_E_2	70	51	54	67	68	56	67	70	59	70	109
80_F_1	66	72	57	47	56	50	64	48	50	64	183
80_F_2	60	62	67	38	55	61	61	48	62	50	160

Showing for each of the 24 arrays from chips 3434238066 and 3434238080 (abbreviated to 66 and 80) the number of pairs of neighbouring beads of the same type arising from 10 permutions. The actual number of observed pairs is also shown.
